# A study protocol for identifying aging trajectories toward chronic neurodegenerative diseases by means of Marche regional administrative databases – TREND project

**DOI:** 10.3389/fpubh.2024.1354538

**Published:** 2024-04-23

**Authors:** Liana Spazzafumo, Jacopo Sabbatinelli, Leonardo Biscetti, Francesco Balducci, Marco Lilla, Deborah Ramini, Angelica Giuliani, Luca Paciello, Giuseppe Rupelli, Marco Pompili, Giuseppe Pelliccioni, Rina Recchioni, Fabiola Olivieri

**Affiliations:** ^1^Scientific Direction, IRCCS INRCA, Ancona, Italy; ^2^Department of Clinical and Molecular Sciences, Università Politecnica delle Marche, Ancona, Italy; ^3^Clinic of Laboratory and Precision Medicine, IRCCS INRCA, Ancona, Italy; ^4^Unit of Neurology, IRCCS INRCA, Ancona, Italy; ^5^Tech4Care srl, Ancona, Italy; ^6^Regional Health Agency of Marche, Ancona, Italy

**Keywords:** neurodegenerative disease, administrative databases, dementia, Parkinson’s disease, geographical information system

## Abstract

**Background:**

People are living longer but an increasing number of older people experience chronicity and disability in the latest years of their life. The Marche region is one of the Italian regions where people live the longest lives; therefore, the number of people with age-related chronic diseases is expected to be at least similar, if not higher, compared to the rest of Italy. The identification of the aging trajectories is of huge interest in the arena of public health. Administrative healthcare databases represent valuable reservoirs for reconstructing the trajectories of aging. Here, we present the protocol for a study (TREND project) aimed to integrate existing administrative databases into a Marche regional dataset in order to estimate the prevalence and incidence rates of age-related neurodegenerative diseases (ND), with a specific focus on Parkinsonism and Dementia.

**Methods:**

The TREND Project is a retrospective cross-sectional study. The source population includes permanent residents in the Marche region aged 40 years and older. A minimal dataset has been built up linking data on drug prescriptions, outpatient services, and diagnosis for hospital admission, from 2014 to 2021 in the Marche Region. Data on clinical outcomes (re-hospitalization, mortality, comorbidities), and therapeutic approaches (drugs and medicines) have been integrated with state-of-the-art statistical methods to define patients into different risk clusters and to analyze the aging trend by assessing the Comorbidity Index (CI) as a proxy for chronicity.

**Discussion:**

Our research contributes to the integration of existing administrative databases on ND to create a Marche regional ND database, support regional health policy, and better understand patients’ needs and their aging trajectories. This approach could be implemented also at the National level. Moreover, by linking different administrative data sources, this study sheds light on important issues related to ND, such as early-onset dementia; ethical aspects such as anticipated wills; problems of dementia in patients still in the job market, etc. The results of this study will contribute to the successful implementation of integrated care for patients affected by ND at regional or national levels.

## 1 Introduction

Despite notable advances in diagnosis and therapeutic management, we are still largely unsuccessful at postponing or preventing the chronic complications of the most common age-related diseases, especially neurodegenerative diseases, including Alzheimer disease (AD) and dementia, and Parkinson’s disease (PD). In other words, we have been becoming more and more able to increase the life expectancy of our patients, but we were not equally able to improve their quality of life. Although the human average life expectancy in developed countries has increased dramatically, this rise has, indeed, been accompanied by an increase in the prevalence of chronic disorders (1), including neurodegenerative diseases (ND), which in turn are the major causes of disability and mortality.

According to the latest WHO data published in 2020, dementia represented 7.64% of total deaths in Italy (2). The prevalence of dementia, considered in all its forms, is estimated at around 9% in the Italian population over 65 years (3). Likewise, PD, which is the second most common neurodegenerative disorder worldwide after AD, has become more and more frequent due to the increasing life expectancy (4). In fact, from 1990 to 2016, the number of patients with PD globally increased to 6.2 million (5). PD is frequently accompanied by dementia, with a point prevalence of 30% (6).

Considering the strict relationship between the increase in life expectancy and the burden of ND in developed countries, research initiatives aimed at increasing health span and compressing morbidity are of crucial clinical and socio-economical relevance. Such complex multimorbidity is a major challenge to existing models of healthcare delivery and there is a need to ensure integrated care across disease pathways and primary and secondary care (7).

From a gerontology/geriatric point of view, the aging process is the result of the combined effects of selective and remodeling forces toward achieving human longevity (8, 9). From a clinical viewpoint, successful aging can be measured as a multidimensional construct that reflects the complexity and dynamics of many physiological systems (10). Perturbations and, hypothetically, a narrowing of this complexity may reduce the ability to adapt to stress and lead to frailty (11). In this framework, the analysis of care trajectories through the analysis of administrative health databases has been proposed as a tool for population-based estimations and is increasingly being adopted to obtain estimates of the aging process, disease burden, quality of care, and resource allocation (12). Healthcare administrative databases show many advantages, such as ease of access, the wide range of tracked diseases and comorbidities, and the ability to provide both cross-sectional and longitudinal data that can be analyzed and visualized also by the use of Geographical Information Systems (GIS) (13). GIS provide a valuable aid in tailoring interventions, optimizing resource allocation, and strategically placing healthcare facilities to address specific chronic health challenges in diverse geographical areas.

The combined analysis of different healthcare administrative datasets has proved useful to healthcare demand in diverse population segments (14). A similar approach was applied to ND using French national administrative databases (15). The prevalence of ND was presumed from drug reimbursement data, hospital stays or registration of a chronic condition, and the different neurological diseases were identified through validated algorithms (15). Here, we will apply a similar approach to estimate the prevalence and incidence of ND in the Marche Region.

The Marche Region is one of the Italian Regions where people live the longest lives, with a higher life expectancy at birth compared to the median one in Italy (81.2 vs. 80.5 years) (16) and a significant representation of older people among residents. Indeed, in the Marche Region, people aged ≥65 years represent over 25% of the total resident population, while in Italy people belonging to this age group are 24.1% of the total residents (16). Based on these epidemiological data, we would expect significant incidence and prevalence rates of ND in the Marche region. In order to make an estimate as precise as possible of ND burden on Marche Healthcare and families, we have drawn up the present study protocol, namely the TREND (Identifying aging TRajEctories toward chronic Neurodegenerative Diseases by means of Marche regional administrative databases) project.

The main aims of the TREND project are (i) to create a minimum dataset from administrative data of Marche residents; (ii) to estimate the prevalence and incidence of neurodegenerative diseases in the Marche Region, also by taking advantage of a GIS; (iii) to stratify the subjects into different risk groups that share common characteristics (demographic assessment, clinical status, comorbidity, drugs, and therapies); (iv) to assess the comorbidity index (CI) as a tool to depict the aging trajectories and discriminate between poor, medium and high risk of unsuccessful aging.

## 2 Methods and analysis

### 2.1 Study design

The TREND Project is a retrospective cross-sectional study. The source population includes permanent residents in the Marche region aged 40 years and older.

The dataset is built up by linking this database with the data on drug prescriptions, outpatient services, exemptions, and diagnosis for hospital discharge collected between 2014 and 2021. Three categories of subjects are identified, i.e., patients with (i) Parkinsonism that includes PD and Atypical Parkinsonism (AP), (ii) AD and non-AD dementia including PDD (subsequently referred to as ‘Dementia’), and (iii) Parkinsonism and Dementia. To exclude the patients with prior ND diagnosis a 5-year period of freedom from disease is deemed as appropriate.

The three groups of patients are identified through validated algorithms analysis of administrative healthcare databases. Moreover, data on clinical outcomes (re-hospitalization, mortality, and comorbidities) and therapeutic approaches (drugs and medicines) are integrated with state-of-the-art statistical methods to define patients into different risk clusters and to measure a “chronicity index.”

#### 2.1.1 Data sources

Data sources include the administrative electronic health archives present in the Marche region in order to trace the subject in all accesses to the health services of interest for the period 2014–2021. Specifically:

- Patient registry: these inhabitants have been identified using the archives of the Regional Population Registry (ARCA) that contain demographic and administrative information. To preserve the individual’s privacy, data are anonymized by removing personal information.
- Outpatient’s drug prescription database reporting all dispensations of drugs reimbursable by the National Health System (NHS) and the list of drugs directly supplied by the hospital pharmacy.
- Hospital Discharge Records (HDR) considering the principal and up to five secondary diagnoses, categorized using the International Classification of Diseases-Ninth Revision, Clinical Modification code (ICD-9), and different precision levels were considered based on the disease.
- Ticket Exemptions, that record information on all co-payment exemptions due to chronic disease.

In addition to these fundamental databases, other linking tables are considered, i.e., the database containing detailed information on drugs (commercial name, ATC and AIC codes, public price, etc.), regional and municipal population (obtained from Istat source), and other data not directly used by the study protocol (e.g., hospital pharmaceuticals).

After matching patient data from various tables based on their social security number, the data was anonymized, assigning each patient a unique ID to ensure irreversibility and prevent any attempt to trace back to the social security number from the corresponding ID.

### 2.2 Data management and quality check

To improve the accuracy of the data in preparation for subsequent analyses, data from all sources have been cleaned, and prepared for analysis by the in-house statistics, bioinformatics and data management teams following main operating procedures:

- Administrative data coming from various tables with different structures and characteristics have been processed to harmonize their structure;
- Inconsistent or incorrect data have been assessed by logical and range checks to address ambiguous values (non-uniform coding, empty values, and missing data);
- The source data in a raw format (i.e., as a single text field in the case of exemptions) have been validated with a normalization procedure.

In addition to data management, a Structured Query Language (SQL) syntax is set up in order to perform quality check procedures.

For each available year, a record count is performed, together with the count of unique patients (distinct identification codes), and several reference fields (e.g., number of prescriptions for pharmaceuticals, number of diagnoses for hospitalizations, etc.). Moreover, missing and invalid values were handled (i.e., by replacing blank text fields, or inconsistent/dummy values). In order to assess the temporal trend of these differences, the overall average for all available years and the study horizon 2014–2021, and the percentage shares on the total number of records and on the total number of patients, are calculated for each table.

### 2.3 Algorithm for subject extraction

First, patients with PD and Dementia will be identified from administrative regional databases records, by filtering according to prescription data, hospital discharge records (HDRs), and medical exemptions due to chronic disease. Based on the Italian guidelines for the diagnosis and treatment of PD (17) and AD (18), tracer drugs for PD and Dementia are identified (Table 1). Specifically, pharmacological treatments for AD (reimbursed by the Italian National Healthcare System) include anticholinesterase drugs and memantine. Nevertheless, the rate of anticholinesterase drug prescription in AD is extremely variable, ranging from 11 to 80% in the geriatric population (19). Moreover, there are no specific drugs for non-AD dementia that are reimbursed by National Health system. Therefore, the estimation of Dementia incidence and prevalence based on specific AD pharmacological therapy prescription is clearly associated with a relevant risk of underestimation. Thus, we have considered the inclusion of further drug classes in the list of tracer active principles. According to extensive literature data, patients with dementia are significantly more likely to be prescribed antipsychotic and antidepressant drugs compared to subjects without dementia (20, 21). Moreover, a previous machine learning analysis of administrative data revealed that, besides anticholinesterase drugs, antipsychotics were the most important predictors of dementia (22). Thus, we included antipsychotic drugs and the antidepressant drugs trazodone and mirtazapine in the list of tracer drugs (Table 1). In this case, to avoid overestimation due to inappropriate inclusion of subjects suffering from primary psychiatric issues, we excluded subjects that were hospitalized for schizophrenia, bipolar disorder, or major depressive disorder or were attributed a ticket exemption for psychoses.

**Table 1:** ATC codes for tracer drugs.

Neurodegenerative disease	ATC code of tracer drugs
Dementia	N06DA02 Donepezil
	N06DA03 Rivastigmine
	N06DA04 Galantamine
	N06DX01 Memantine
	N05AA01 Chlorpromazine
	N05AA02 Levomepromazine
	N05AA03 Promazine
	N05AD01 Haloperidol
	N05AH03 Olanzapine
	N05AH04 Quetiapine
	N05AL05 Amisulpride
	N05AX08 Risperidone
	N05AX09 Clotiapine
	N05AX12 Aripiprazole
	N06AX05 Trazodone
	N06AX11 Mirtazapine
Parkinsonism	N04BA02 Benserazide/levodopa
	N04BA03 Carbidopa/levodopa/entacapone
	N04BA05 Melevodopa/carbidopa
	N04BA06 Melevodopa/carbidopa
	R05DB27 Levodropropizine
	N04BC04 Ropinirole
	N04BC05 Pramipexole
	N04BC07 Apomorphine
	N04BC09 Rotigotine
	N04BD01 Selegiline
	N04BD02 Rasagiline
	N04BD03 Safinamide
	N04BX01 Tolcapone
	N04BX02 Entacapone
	N04BX04 Opicapone

HDRs have been analyzed to extract subjects that had an in-hospital stay during the observation period for the diagnostic work-up and management of PD and dementias. The list of ICD-9 codes selected for the disease tracing has been drawn up to be as comprehensive as possible, following the different interpretations of the guidelines for compiling the HDRs adopted by the hospitals operating in the area. For example, although the indication to report the ICD-9 diagnosis code as specifically as possible (e.g., 290.0) is in force, in many cases the HDR is filled with the parent code of the diagnosis tree (e.g., 290). The tracer ICD-9 codes for each group are listed in Table 2.

**Table 2:** ICD-9 codes for the identification of subjects with dementia.

Neurodegenerative disease	ICD-9 codes
AD	331.0 –Alzheimer’s disease
Non-AD dementia	290 – Dementias 290.0 – Uncomplicated senile dementia 290.1 – Pre-senile dementia 290.10 – Uncomplicated pre-senile dementia 290.11 – Pre-senile dementia with delirium 290.12 – Pre-senile dementia with delusional aspects 290.13 – Pre-senile dementia with depression aspects 290.2 – Senile dementia with delusional or depression aspects 290.20 – Senile dementia with delusional aspects 290.21 – Senile dementia with depression aspects 290.3 – Senile dementia with delirium 290.4 – Vascular dementia 290.40 – Uncomplicated vascular dementia 290.41 – Vascular dementia with delirium 290.42 – Vascular dementia with delusions 290.43 – Vascular dementia with behavioral disturbance 294.1 – Dementia in other diseases classified elsewhere^*^ 294.10 – Dementia in other diseases classified elsewhere, without behavioral disturbances^*^ 294.11 – Dementia in other diseases classified elsewhere, with behavioral disturbances^*^ 331.1 – Frontotemporal dementia 331.11 – Pick’s disease 331.19 – Other frontotemporal dementias 331.2 – Senile degeneration of brain 331.3 – Communicating hydrocephalus 331.5 – Normal pressure hydrocephalus 331.7 – Degeneration of brain in other diseases classified elsewhere 331.82 – Dementia with Lewy bodies 331.9 – Brain degeneration, unspecified

^*^Patients with either of the codes 294.1, 294.10, or 294.11 should not be included in the non-AD group if they also have the code 331.0 (Alzheimer’s disease) among the diagnoses. Conversely, if one or more of these codes are not associated with the 331.0 code, the patient should be included in the non-AD group.

To identify subjects with neurodegenerative disease based on prescription charge exemption due to chronic disease (Exemptions), the codes in Table 3 are applied.

**Table 3:** Exemption codes.

Neurodegenerative disease	Exemption codes
PD	038 – Parkinson’s disease and other extrapyramidal diseases
AD	029 – Alzheimer’s disease
Non-AD dementia	011 – Dementia, all subcodes except 011.291.1 (alcohol amnesic syndrome) and 011.294.0 (nonalcoholic amnesic syndrome)

**Figure 1:**
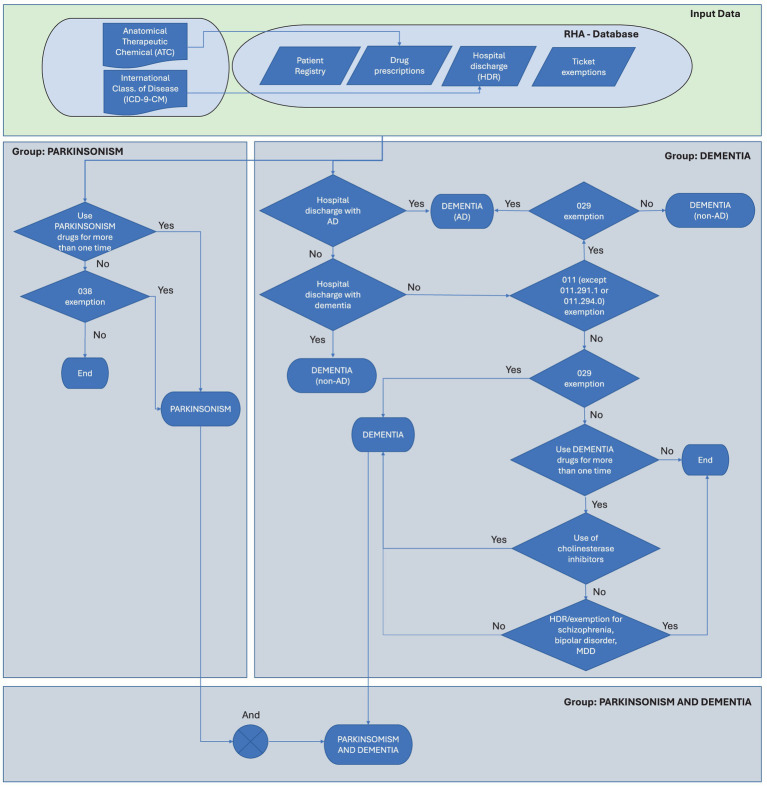
Flow diagram for the adjudication of cases into the Parkinson’s and Dementia groups. HDR, hospital discharge record.

After the identification and extraction of subjects according to ATC/ICD-9/exemption tracer codes, we have proceeded as follows to estimate the prevalence of subjects in each group. First, occasional PD and Dementia drug users, defined as subjects receiving a single prescription of a tracer drug during the observation period, are identified, and removed. In identifying cases of PD, the use of ICD-9 codes listed in HDRs poses some limitations as they often fail to distinguish among Parkinson’s disease, atypical parkinsonism, and tremor syndromes (23). Therefore, PD patients (Group 1) are identified according to the criterion of either the non-occasional use of PD tracer drugs (listed in Table 1) or the presence of the exemption code ‘038’. According to this definition, patients with early-stage disease may be undetectable. Then, we proceeded by identifying patients with Dementia (Group 2), based on the possession of at least one of the following requirements: (i) non-occasional presence of ATC tracing codes for AD or non-AD dementia (Table 1); (ii) specific diagnosis reported in HDR of either AD (as a primary and secondary diagnosis) or any other form of dementia (Table 2); (iii) attribution of one of the exemption codes listed in Table 3 for dementia; (iv) in presence of non-occasional consumption of antipsychotic drugs, trazodone and/or mirtazapine as the only fulfilled criterion, absence of hospitalization for schizophrenia (ICD-9 code 295.xx), bipolar disorder (296.0x, 296.1x, 296.4x–296.9x), or major depressive disorder (296.2x, 296.3x, 311.xx), and absence of the exemption code ‘044,’ which identifies psychoses. The following step consists in the adjudication of retrieved subjects into the dementia group based on the scheme reported in Figure 1.

In order to operationalize the algorithm above (described in Figure 1), an SQL procedure is performed, involving a series of steps for data extraction, linkage, and analysis:

(i) Identification of tracking drugs according to the ATC codes listed in the protocol and matching them with the AIC codes present in the pharmaceutical flows through the linking database;
(ii) Extraction from the pharmaceutical flows of the records corresponding to the tracer drugs and the selected exemption codes;
(iii) Identification of subjects with selected exemption from the exemption database;
(iv) Integration of the inclusion criteria on HDR diagnoses (for the dementia group only);
(v) Identification of diagnoses and extraction of the corresponding subjects;
(vi) Construction of an analytic database per patient (where each record corresponded to a unique anonymous ID), containing the selection criteria and group classification;
(vii) Creation of a demographic database according to the project requirements (age, sex, mortality, and residential area). Association (linkage) of the medical record to patients according to the anonymous identifiers.
(viii) Construction of automated count tables for each of the groups and subgroups.
(ix) Construction of algorithms for automated calculation of prevalence and incidence (comprehensive at regional aggregate level) for the three groups.

### 2.4 Data visualization platform and GIS

**Figure 2:**
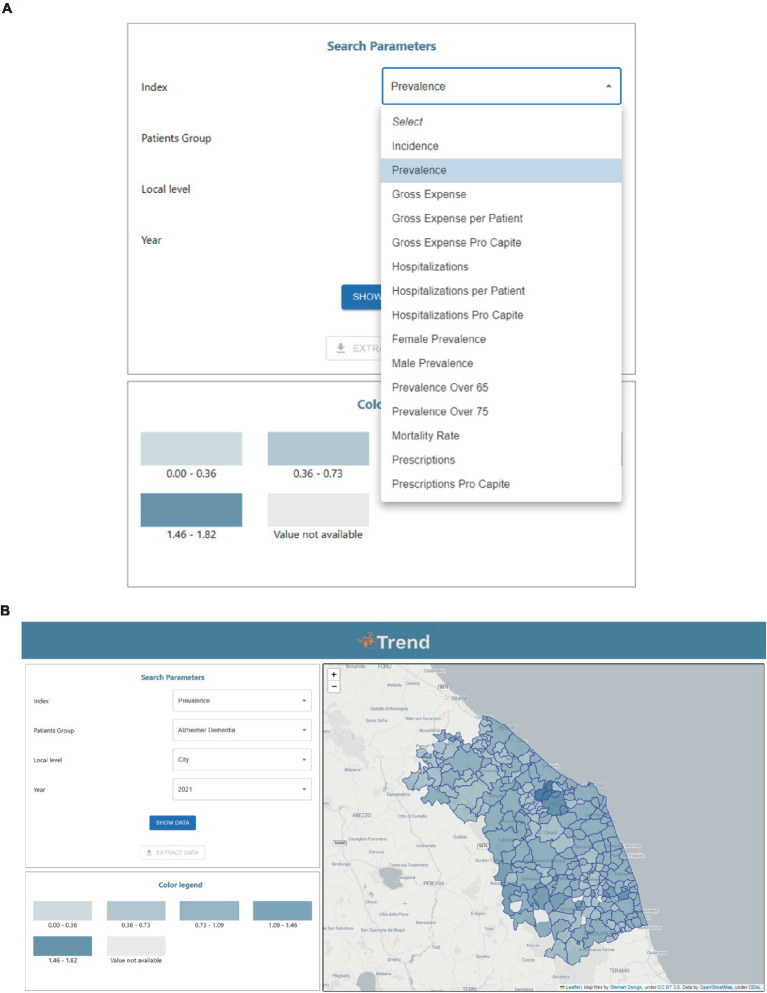
Representative screenshots of the Geographical Information System (GIS) user interface. **(A)** Search parameters, with the index selection tool expanded, **(B)** output of the prevalence of Dementia in 2021 grouped at the municipality level.

The data visualization tool will consist of a web interface, divided into two areas. The first one is for configuring the filters (Figure 2A), and the second one is for presenting the output data (Figure 2B). In addition, we have developed a geographical information system (GIS) to support the regional health policy and to present how the epidemiological measures can be visualized by users, either professionals, politicians or citizens, in an easy and effective way.

In order to better understand how the platform works, a description of the filters is provided below.

- Indicator. The first filter is called “indicator” and refers to which indicator we are interested in visualizing. The available indicators are incidence, prevalence (global, sex-specific, for subjects aged >65 and >75 years), gross expense (total, per patient, pro-capita), hospitalizations (total, per patient, pro-capita), mortality rate, prescriptions (total and pro-capita);
- Patient cohort. The patient cohort filter allows to choose for which cohort of patients we want to visualize data: patients with PD or dementia;
- Local Level. The local level indicates at which level the data should be aggregated. In the TREND database, data are stored at the municipality level, but users may consider as much relevant to explore data aggregated at the province or regional level;
- Year. This filter allows setting the year we are interested in visualizing data for.

As described above, the remaining area is dedicated to data visualization, which can be done in two different modalities according to which level of data aggregation has been selected at the filter “Local level”:

- For “municipality” and “province” levels, the data will be represented, for a specific year, in the form of a map in which each area is colored with a different shade of color depending on the percentile of the selected indicator;
- For the “region” level, the data will be presented in a table in which each row will report the corresponding data of a specific year.

### 2.5 Assessment of comorbidities

To identify clusters of comorbidities in patients with ND, the Comorbidity Index (CI) is computed for each patient with Parkinsonism or Dementia. The presence of the comorbidities included in the CI is inferred based on hospital discharge records and ticket exemptions due to chronic disease. We have calculated the Charlson Comorbidity Index (CCI) for medical conditions by the ICD-9 retrieved from the publication of Glasheen et al. (24). Table 4 lists the CCI comorbidities, the tracer ICD-9, and exemptions codes. Patients will be categorized into low risk (no comorbidities), intermediate risk (1 comorbidity), and high risk (2+ comorbidities).

**Table 4:** Tracer exemption and hospital discharge record (HDR) ICD-9 for each medical condition included in the Charlson Comorbidity Index (CCI).

		CCI points	Exemption code	HDR ICD-9
1	Myocardial infarction	1	–	410, 412
2	Congestive heart failure	1	021	398.91, 402.01, 402.11, 402.91, 404.01, 404.03, 404.11, 404.13, 404.91, 404.93, 425.4, 425.5, 425.6, 425.7, 425.8, 425.9, 428.
3	Peripheral vascular disease	1	0C02	093.0, 437.3, 440, 441, 443.1, 443.2, 443.8, 443.9, 447.1, 557.1, 557.9, V43.4
4	Cerebrovascular disease	1	0B02	362.34, 430, 431, 432, 433, 434, 435, 436, 437, 438
5	Dementia	1	011	290, 290.1, 290.2, 290.3, 290.4, 294, 294.1, 294.2, 294.8, 331, 331.1, 331.2, 331.7, 797
6	Chronic pulmonary disease	1	024 or 057	490, 491, 492, 493, 494, 495, 496, 500, 501, 502, 503, 504, 505, 506.4, 508.1, 508.8
7	Rheumatic disease	1	006 or 067	446.5, 710, 710.1, 710.2, 710.3, 710.4, 714, 714.1, 714.2, 714.8, 725
8	Peptic ulcer disease	1	-	531, 532, 533, 534
9	Hemiplegia or paraplegia	2	-	334.1, 342, 343, 344
10	Renal disease	2	023	403.01, 403.11, 403.91, 404.02, 404.03, 404.12, 404.13, 404.92, 404.93, 582, 583, 583.1, 583.2, 583.3, 583.4, 583.5, 583.6, 583.7, 585, 586, 588, V42.0, V45.1, V56
11	Liver disease, mild	1	016	070.22, 070.23, 070.32, 070.33, 070.44, 070.54, 070.6, 070.9, 570, 571, 573.3, 573.4, 573.8, 573.9, V42.7
	Liver disease, moderate to severe	3	008	456, 456.1, 456.2, 572.2, 572.3, 572.4, 572.8
12	Diabetes without chronic complications	1	013	250.8, 250.9, 249, 249.1, 249.2, 249.3, 249.9
	Diabetes with chronic complications	2	013	250.4, 250.5, 250.6, 250.7
13	Any malignancy	2	048	14x., 15x., 16x., 170, 171, 172, 174, 175
	Metastatic solid tumor	6	048	196, 197, 198, 199
14	HIV infection, no AIDS	2	020	042
	AIDS	6	020	112, 180, 114, 117.5, 007.4, 078.5, 348.3, 054, 115, 007.2, 176, 200, 201, 202, 203, 204, 205, 206, 207, 208, 209, 031, 136.3, V12.61, 046.3, 003.1, 130, 799.4, 010, 011, 012, 013, 014, 015, 016, 017, 018

### 2.6 Statistical analysis

After extracting patients affected by Parkinsonism and Dementia over the period 2014–2021, all entries retrieved in each year of the observation period will be used for calculating prevalence and incidence rates for each year. The incident cohort represents the proportion of individuals in which the neurodegenerative pathology has manifested during the inclusion period. Subjects are identified through their presence in at least one of the archives considered in the recruitment year and the simultaneous absence in all the archives of interest in the 5 years preceding the one under consideration. Each subject will be identified by its own unique anonymous code across all data sources.

A descriptive analysis of the patients will be conducted using uni- and bi-variate statistical analyses, with the aim of verifying the comparability of different risk groups for ND that share common characteristics (demographic assessment, clinical status, drugs, and therapies). Continuous variables will be expressed using means with standard deviations (SD) and medians with ranges. For variables with a normal distribution, statistical comparisons among groups will be made using an analysis of variance test. Measurements with discrete distribution will be expressed as percentages (%) and analyzed by the Chi-squared or Fisher’s exact test when appropriate.

Cluster analysis will be applied to stratify patients affected by ND into different risk groups of “unsuccessful aging.” Discriminant function analysis will be performed using both forward and backward stepwise algorithms on each cluster model to evaluate the input variables that will be significant determinants of model structure, using the hierarchical clustering approach. The complete linkage criterion will be used to measure the distances between clusters because it leads to the best result. An advantage of hierarchical clustering on variables is that the outcome can easily be represented by means of a dendrogram. The groups will be identified based on the following dimensions: age, gender, ND, comorbidities, medications, and rehospitalization.

The distribution of the CI in patient cohorts will be evaluated according to year, sex, and age classes.

The aging trajectories will be reconstructed by applying a regression model to depict the age-related trend of the risk profiles of patients. The regression model will include variables or sets of variables forced into the equation at each step and only the final models will be presented with all variables entered simultaneously. Logistic and survival regression analysis will be applied to calculate the risk of death, hospitalization, and onset of other diseases considering the following covariates: age, sex, groups of pathologies (Dementia vs. PD), number of medications taken, types of medications, frequency and duration of re-hospitalizations and CI. Aging trajectories will be constructed by analyzing the estimated values from logistic and survival regression analyses with patients’ ages based on risk and disease clusters (Dementia vs. PD).

Performances of model approaches will be tested with simulated scenarios and real-world data using the risk stratification cohorts of the present study. Simulations will be generated under a number of scenarios accounting for different patterns of aging of different risk groups of neurodegenerative diseases.

STATA ver.18 (StataCorp, College Station, TX, USA) and R version 4.2.2 (R Core Team) software will be used to perform data analyses.

## 3 Discussion

Dementia is one of the major health challenges for our generation, with close to 50 million people affected worldwide. Dementia is currently the seventh leading cause of death and one of the major causes of disability and dependency among older people globally so that the personal, social, and economic consequences of dementia are enormous. In recent years, healthcare research in planning care strategies for dementia has received increasing attention (25). Healthcare administrative databases are increasingly employed to improve care transitions between different settings (Family Medicine Groups, home care, and community services) and the care of people living with dementia and their caregivers (26). Efforts are needed to monitor the incidence and progression of neurodegenerative diseases also in our Country, and Italian administrative databases offer an opportunity for the innovative generation of information. Indeed, archives of the National Health Service that provide demographic and administrative information can be linked with archives of ticket exemptions, hospital discharge, and drug prescriptions to estimate the prevalence of subjects affected by specific conditions and to stratify the population according to such conditions (27, 28).

Attention to local and regional needs for neurodegenerative diseases is crucial for organizing services in the framework of National Laws and assuring basic levels of quality care (LEA). Our research may contribute to reaching these goals through the integration of existing administrative databases on ND in order to create a Marche Regional ND dataset, that could be implemented at the National level. The identification of subjects affected by a specific disease through access to healthcare services starting from drug prescription data registered in an administrative healthcare archive has the advantage of using codified, standardized, and quality-controlled definitions. It also represents a point in the natural history of the disease that is easily identifiable and applicable to all the individuals considered, easily and homogeneously identifying the onset of the disease. Despite minimal regional variations in the process of data collecting, our protocol is feasible for the immediate application also in other Italian regions. Indeed, computer-based gathering of hospital discharge records and drug reimbursement data is in force in all the Italian regions, and the exemption codes that were considered in the present protocol are applicable throughout the entire National healthcare system. Despite the peculiarities of the Italian healthcare system, it remains evident that studying algorithms proposed internationally also provides valuable insights into the selection of criteria, especially concerning medication criteria and diagnostic codes for hospital stays. The methodology presented in this protocol may be easily adapted to other countries, including healthcare systems based on insurance, as universal ICD-9 and ATC codes are utilized for retrieving cases, which are commonly available across various healthcare systems. The possible underestimation of the individuals number affected by the pathological condition of interest (limited sensitivity) is compensated by identified patients who reasonably present the pathological condition of interest (good specificity), being negligible the probability that a subject not affected by the pathology will access specific health services.

Existing administrative databases are readily available and cost-effective and allow for the analysis of large population samples, providing statistical power to detect trends and patterns in ND incidence and prevalence. Possible limitations of this approach may include selection bias, misclassification errors inherent in retrospective data collection, limited completeness of administrative data, which may vary across different healthcare settings, and the inability to capture detailed clinical information that may be available in prospective registries that may, on turn, suffer from recruitment bias and required challenging and time-consuming follow-up procedures (29).

Overall, based on the data collected in Marche regional healthcare administrative databases on PD and dementia, it will be possible to calculate the prevalence rates for each year, reconstruct the aging trajectories, depict the age-related trends of the risk profiles of patients, and provide geographical information on ND patients in Marche region. The results of this project will support regional health policy planning and contribute to the successful implementation of integrated care for patients affected by ND at the regional and national levels.

## Ethics statement

As required by the Ministry of Health, this retrospective study was notified to the Marche Region Ethics Committee. Written informed consent to participate in this study was not required from the participants or the participants’ legal guardians/next of kin in accordance with the national legislation and the institutional requirements.

## Author contributions

LS: Conceptualization, Data curation, Funding acquisition, Project administration, Writing – original draft, Writing – review & editing. JS: Formal analysis, Investigation, Methodology, Writing – original draft, Writing – review & editing. LB: Methodology, Validation, Writing – review & editing. FB: Data curation, Formal analysis, Software, Writing – review & editing. ML: Formal analysis, Software, Writing – review & editing. DR: Methodology, Writing – review & editing. AG: Validation, Writing – review & editing. LP: Investigation, Software, Visualization, Writing – review & editing. GR: Resources, Supervision, Writing – review & editing. MP: Data curation, Resources, Writing – review & editing. GP: Methodology, Writing – review & editing. RR: Resources, Writing – review & editing. FO: Funding acquisition, Supervision, Writing – original draft, Writing – review & editing.
